# Secretagogin is increased in plasma from type 2 diabetes patients and potentially reflects stress and islet dysfunction

**DOI:** 10.1371/journal.pone.0196601

**Published:** 2018-04-27

**Authors:** Sara F. Hansson, Alex-Xianghua Zhou, Paulina Vachet, Jan W. Eriksson, Maria J. Pereira, Stanko Skrtic, Helen Jongsma Wallin, Anders Ericsson-Dahlstrand, Daniel Karlsson, Andrea Ahnmark, Maria Sörhede Winzell, Maria Chiara Magnone, Pia Davidsson

**Affiliations:** 1 Translational Science, Cardiovascular, Renal and Metabolism, IMED Biotech Unit, AstraZeneca, Gothenburg, Sweden; 2 Department of Medical Sciences, Clinical Diabetes and Metabolism, Uppsala University, Uppsala, Sweden; 3 Translational Medicine Unit CVRM, Early Clinical Development, IMED Biotech Unit, AstraZeneca, Gothenburg, Sweden; 4 Institute of Medicine at Sahlgrenska Academy, University of Gothenburg, Gothenburg, Sweden; 5 Offspring Biosciences, Södertälje, Sweden; 6 Bioscience, Cardiovascular, Renal and Metabolism, IMED Biotech Unit, AstraZeneca, Gothenburg, Sweden; Children's Hospital Boston, UNITED STATES

## Abstract

Beta cell dysfunction accompanies and drives the progression of type 2 diabetes mellitus (T2D), but there are few clinical biomarkers available to assess islet cell stress in humans. Secretagogin, a protein enriched in pancreatic islets, demonstrates protective effects on beta cell function in animals. However, its potential as a circulating biomarker released from human beta cells and islets has not been studied. In this study primary human islets, beta cells and plasma samples were used to explore secretion and expression of secretagogin in relation to the T2D pathology. Secretagogin was abundantly and specifically expressed and secreted from human islets. Furthermore, T2D patients had an elevated plasma level of secretagogin compared with matched healthy controls, which was confirmed in plasma of diabetic mice transplanted with human islets. Additionally, the plasma secretagogin level of the human cohort had an inverse correlation to clinical assessments of beta cell function. To explore the mechanism of secretagogin release *in vitro*, human beta cells (EndoC-βH1) were exposed to elevated glucose or cellular stress-inducing agents. Secretagogin was not released in parallel with glucose stimulated insulin release, but was markedly elevated in response to endoplasmic reticulum stressors and cytokines. These findings indicate that secretagogin is a potential novel biomarker, reflecting stress and islet cell dysfunction in T2D patients.

## Introduction

Type 2 diabetes mellitus (T2D) is the most common form of diabetes [1,2]. One of the key pathological mechanisms for the disease progression is the gradual loss of pancreatic beta cells. It leads to insufficient release of insulin to compensate for the insulin resistance in tissues and thus an impaired glucose homeostasis in the body [3]. The major defect leading to a decrease in beta cell mass in T2D is increased apoptosis, while new islet formation and beta cell replication are less affected [4]. Multiple stress conditions, including endoplasmic reticulum (ER) stress [5–7] and enhanced production of pro-inflammatory cytokines [8,9], are present in the T2D islets and cause beta cell dysfunction and apoptosis. Chronic activation of biological systems involved in emotional or social stress response, could contribute to ER stress and ultimately beta cell functions, such as endothelial dysfunction [10], changes in energy pathways [11], or cellular mechanisms [12–14]. Synergistic effects of stress together with exposure to eugenic agents may further enhance activation of biological systems involved in stress response, such as dysfunctional cell division processes [15].

The current gold standard biomarkers of T2D include fasting plasma glucose, oral glucose tolerance test (OGTT), HbA_1c_, and biomarkers of beta cell capacity for insulin release, such as insulin and C-peptide. However, these biomarkers do not provide a comprehensive picture of the status of islet cells, for instance overall mass, viability or ability to cope with micro-environmental stressors at the islet level [16]. The study of proteins released specifically from the islets and their relation to islet cell stress could provide a source of novel biomarkers reflecting the islet status. Consequently, they may allow a more informed decision-making on novel therapeutic approaches aiming at restoring beta cell function and islet health.

Secretagogin is a protein consisting of six EF-hand calcium-binding domains [17]. It is highly expressed at mRNA level in human islets [17,18] and at protein level in the rat endocrine pancreas [19,20]. Secretagogin is also expressed by a subpopulation of endocrine cells of the gastrointestinal tract and neuronal cells [17], particularly in cerebellum, pituitary gland and hypothalamus [21]. Through systematic comparison of the expression in multiple human tissues, the Human Protein Atlas displays that secretagogin has its highest protein expression level in islets of Langerhans, compared with other tissues [22].

The precise physiological role of secretagogin is not yet known. However, previous studies have shown that it is involved in calcium influx, diminished cell proliferation [10], improved insulin secretion [17,23,24], beta cell homeostasis [25], apoptosis [26] as well as transcription and control of insulin secretion [27]. A recent study by Malenczyk and co-workers [28] showed that secretagogin knockout mice developed progressive glucose intolerance and loss of beta cells with induced ER stress. Their study confirmed interaction of secretagogin with the cytoskeleton and exocytosis machinery and proposed that intracellular secretagogin promotes pancreatic beta cell survival and decreases ER stress by stabilizing deubiquitinating proteins [28]. On the other hand, these previous functional studies of secretagogin were performed in animal islets, animal beta cell lines or rodent *in vivo* models. It raises questions regarding their translatability, given the important differences between human and rodent islets [29]. Nevertheless, no studies of secretagogin release from primary human islets and human beta cells have been reported previously.

The present study aims to assess secretagogin as a potential soluble biomarker of human islets stress by using translational *in vivo* and *in vitro* models and determining the secretagogin level in plasma samples from diabetes patients compared with healthy controls.

## Materials and methods

### Cohort of study

The clinical samples were from two merged cohorts and consisted in total of 26 T2D and 26 healthy control subjects (Table 1). The first cohort of 20 T2D and 20 healthy controls matched for gender, age and BMI has previously been described by Pereira *et* al. [30]. The second cohort is an addition, by six individuals per group, from the same clinical site using a similar but reduced clinical protocol. The additional subjects were also fasted overnight, but in this instance fasting blood samples were collected at only one occasion, without performing oral glucose tolerance test (OGTT) or metabolic imaging. The clinical and biochemical characteristics measured are given in the result section, Table 1.

**Table 1:** Clinical and biochemical characteristics of study participants and correlations between characteristics and the secretagogin (SCGN) level.

	Control	T2D	T2D vs. Ctrl (p)	Corr. with SCGN
Number of participants (N)	26 (13 F, 13 M)	26 (12 F, 14 M)	N/A	---------
Age (years)	58 ± 12	60 ± 9	0.9	r = 0.13, p = 0.4
BMI (kg/m2)	30.1 ± 4.6	29.8 ± 4.5	0.7	**r = -0.30, p = 0.04**
HbA1c (%)	5.49 ± 0.36	6.77 ± 1.02	**<0.0001**	**r = 0.46, p = 0.04**
HbA1c (mmol/mol)	36.5 ± 3.96	50.5 ± 11.1	**<0.0001**	**r = 0.46, p = 0.04**
Plasma glucose (mmol/L)	6.00 ± 0.71	8.60 ± 2.48	**<0.0001**	**r = 0.45, p = 0.001**
Plasma insulin (mU/L)	10.9 ± 5.66	13.8 ± 6.21	0.06	r = 0.05, p = 0.7
Serum C-peptide (nmol/L)	0.88 ± 0.34	1.06 ± 0.32	**0.03**	**r = 0.30, p = 0.03**
Plasma Proinsulin (pmol/L)	11.8 ± 7.54	26.0 ± 18.1	**0.0001**	r = 0.25, p = 0.7
Plasma Proinsulin/C-peptide (pmol/L)/ (nmol/L)	13.0 ± 4.55	24.0 ± 16.3	**0.0002**	r = 0.17, p = 0.2
Plasma Secretagogin (pg/mL)	60.7 ± 36.0	103 ± 58.1	**0.0037**	---------
Plasma Glucagon (pmol/L)	4.26 ± 2.33	5.70 ± 3.72	**0.02**	r = 0.045, p = 0.8
HOMA IR	2.92 ± 1.67	5.13 ± 2.85	**0.0009**	**r = 0.30, p = 0.03**
HOMA2%B	59.9 ± 14.0	39 ± 13.8	**<0.0001**	**r = -0.41, p = 0.003**
Insulinogenic index^¤^	33.4 ± 14.0	5.60 ± 4.64	**0.0004**	**r = -0.56, p = 0.0002**

Data are mean ± SD. Differences between groups were measured with Mann–Whitney U test.

Correlations between secretagogin and other markers were assessed by the Spearman correlation coefficient.

Bold values indicate statistical significance (p<0.05).

Blood chemistry is fasting.

T2D, type 2 diabetes; F, females; M, males. BMI, Body mass index; HbA1c, glycated hemoglobin

¤ A subset of the cohort analyzed (Control, n = 20; T2D, n = 20)

All T2D subjects were on a stable dose of metformin for at least the past three months as their only anti-diabetic medication. Subjects with type 1 diabetes, endocrine disorders, cancer or other major illnesses were excluded, as well as those having ongoing medication with systemic glucocorticoids, beta blockers or immune modulating therapies. The studies were approved by the Regional Ethics Review Board in Uppsala and all participants gave their written informed consent (Ethical approval number, 2013/183 and 2013/494).

### Human pancreatic tissue

For the two-dimensional gel analysis pancreatic tissue and islets, not used for transplantation, was obtained from multi-organ donors with informed consent from the organ donor registry or from relatives. Approval for conduction of the present study was obtained from the local Ethics Committee at Uppsala University, Uppsala, Sweden. Human pancreatic islets were isolated at Uppsala University as described previously [31,32]. Human pancreatic tissue used for immunohistochemical analysis (donor information in Table 2) and primary islets used for xenograft transplantation were also purchased from Prodo Laboratories Inc. (Irvine, CA, USA), providing donor pancreas obtained from deceased individuals with research consent from Organ Procurement Organizations (OPOs). The use and storage of human islets and tissue samples were performed in compliance with the Declaration of Helsinki, ICH/Good Clinical Practice and was approved by the independent Regional Ethics Committee. Primary human islets, from seven donors were incubated in Prodo islet medium-standard (PIM-S) complete medium containing 5.5mM glucose, under humidified atmosphere with 5% CO_2_ at 37°C for 24h. Islet culture media were collected and centrifuged at 4°C, 2000×g for 10 minutes and levels of secretagogin was measured using ELISA (BioVendor).

**Table 2:** Demographic information of the donors of pancreatic tissue used for immunohistochemical analysis.

	Control	T2D	*p*
N (Males, Females)	8 (6 M, 2 F)	8 (6 M, 2 F)	N/A
Age (years)	45 ± 14	50 ± 8	0.27
BMI (kg/m^2^)	30.7 ± 7.1	30.1 ± 6.6	0.88
HbA1c (%)	5.6 ± 0.3	7.4 ± 0.8	**0.0007**

Data are mean ± SD. Differences between groups were measured with Mann–Whitney U test.

Bold values indicate statistical significance (p<0.05).

T2D, type 2 diabetes; F, females; M, males. BMI, Body mass index; HbA1c, glycated hemoglobin.

### Animals and human islet transplantation procedure

All animal procedures were performed following the guide for the care and use of laboratory animals, eight edition, under experimental protocols approved by the Local Ethics Review Committee on Animal Experiments (Gothenburg region). The AstraZeneca R&D Gothenburg animal vivarium is accredited by the Association for the Assessment and Accreditation of Laboratory Animal Care (AAALAC). Immune-compromised male NUDE (Crl:NMRI-Foxn1nu) mice (Charles River, Germany), 6 weeks of age at arrival, were single housed in autoclaved, individually ventilated cages on a 12 hour light/dark cycle (lights on at 6 am) with free access to chow diet (R70, Lantmännen, Sweden) and water ad lib. Three days before the transplantation mice were made diabetic by a single intravenous injection of Alloxan monohydrate (72.5 mg/kg, Sigma Aldrich, UK) dissolved in saline. Mice with blood glucose >20 mM were included in the study. Body weight (26.7±1.3g at start) were monitored three times per week throughout the study. Individuals that showed signs of affected health condition, or decreased 10% or more of its initial body weight during the study were excluded according to the ethical experimental protocol.

Human pancreatic islets were purchased from Prodo Laboratories Inc., USA. Before transplantation the islets were allowed to recover for 24h in complete Prodo Islet Media Standard PIM(s). Human islets were prepared into islet suspension of 1000 adult human islet equivalents (IEQs)/mouse in cold saline. Islets were loaded into a catheter (P50) and kept on cold until the transplantation. The surgical procedure was performed under aseptic conditions. The mouse was anesthetized with isoflurane, washed and wiped with chlorhexidine solution. A small cut was made in the skin to expose the kidney. The kidney capsule was scratched by a needle and a glass rod was used to make a pocket under the kidney capsule. The P50 catheter containing the islets was inserted in the pocket and the islets were slowly injected. After the surgery, the mice were given analgesia (Temgestic, 0.06 mg/kg one dose immediately after surgery and a second dose the day after) and were allowed to wake up on a warm pad and then transferred back to the home cage. Seven mice were transplanted with human islets from one donor. The function of the grafted islets was followed for 38 days by regular measurement of blood glucose (AccuCheck) and circulating human c-peptide levels (Mercodia, Uppsala, Sweden). At termination, blood samples were collected and plasma was separated and stored frozen until ELISA analyses were performed.

### ELISA analyses

A human secretagogin ELISA (BioVendor, Brno, Czech Republic) was used. The plates were blocked with a solution of membrane blocking agent (1%, GE Healthcare) and Rotiblock (1× dilution, Carl Roth GmbH), rinsed with provided kit wash and the manufacturer’s instructions were followed. The plasma samples were diluted three-fold, media samples five-fold, and EndoC extracts 100-fold, prior to analysis. Insulin, human C-peptide and proinsulin ELISAs were used for measurements in plasma, according to the manufacturer’s instructions (Mercodia, Uppsala, Sweden). All samples were assayed in duplicates, randomized and blinded during the run. All ELISA results were obtained using a microplate reader (SpectraMax M2, Molecular devices, Ca, USA).

### Proteomic analysis with two-dimensional gel analysis and mass spectrometry

Proteins were extracted from human islets and exocrine pancreas followed by protein precipitation and analysis with differential in gel electrophoresis (DIGE, GE-healthcare). Selected 2D gel spots were subjected to in gel tryptic digestion and analyzed using on line nanoflow liquid chromatography (LC) on a hybrid linear ion trap-FT-ICR mass spectrometer equipped with a 7T ICR magnet (LTQ-FT, Thermo Electron, Bremen, Germany). MS/MS data was analyzed using Thermo Proteome Discoverer (Thermo Scientific) with the Mascot search engine (Matrix Science, London, UK) and the UniProtKB/Swiss-Prot 55.3 database (selected for Homo sapiens, 19372 entries and trypsin as enzyme). Further details of the proteomic method are provided in the supporting information (S1 Materials and Methods).

### Western blotting

Protein extracts (10 μg, BCA protein assay kit, Pierce, Life Technologies) and proteins precipitated (ProteoExtract, Calbiochem, Merck Chemicals) from conditioned islet media were separated by SDS-PAGE on NuPAGE® 4–12% Bis-Tris Gels using MES SDS buffer (Life Technologies). The proteins from the gels were transferred using the iBlotsystem (Life Technologies) at 23 V, 6 min. For immunoblotting of secretagogin polyvinylidene fluoride (PVDF) iBlot membranes were used and the membranes were blocked in 1:1 mixture of membrane blocking agent (1%, GE Healthcare) and Rotiblock (1 × Carl Roth GmbH, Karlsruhe, Germany) solution and then incubated for 1h at RT with anti-Secretagogin, rabbit polyclonal ab (HPA006641, Sigma Aldrich) diluted 1:1000 in PBS Tween 0.1% (PBS-T, Sigma Aldrich). After washing, the Goat Anti-rabbit IgG, alkaline phosphatase labeled, secondary antibody (Pierce, Thermo Scientific) was added (1:5000) to the membrane. Immunoreactive bands were visualized using 1-step NTB/BCIP substrate (Pierce, Thermo Scientific). For detection of CHOP, fluorescent western blot with the Odyssey CLx imaging system (LI-COR biotechnology, NE, USA) was used. Proteins from the gels (10μg EndoC cell extract/lane) were transferred to nitrocellulose iBlot membranes. The membranes were blocked in Odyssey blocking buffer (TBS) (LI-COR Biotechnology, NE, USA) and then incubated with anti-CHOP, mouse monoclonal ab (# ab 11419 [9C8], Abcam, Cambridge, MA, USA) diluted 1:1000 in blocking buffer (TBS)-Tween 0.1% at 4°C over night. After rinses in TBS-T membranes were incubated with secondary antibody (donkey anti-mouse 800CW (Odyssey, LI-COR Biotechnology)) diluted 1:15000 in blocking buffer TBS-T for 1h. After extensive washes in TBS-T and then in TBS the membranes were scanned using the Odyssey CLx imager with the Image Studio software, v. 4.0 and the 800 channel selected (LI-COR Biotechnology).

### Quantitative immunohistofluorescence analysis

Formalin fixed and paraffin embedded (FFPE) human pancreatic tissue samples from human normal controls (n = 8) and T2D subjects (n = 8) (Table 2) were sectioned at 4μm thick sections and mounted on glass slides.

An immunohistofluorescence (IHF) assay for simultaneous visualization of insulin, glucagon and secretagogin was developed. The IHF staining process included an initial heat-induced antigen retrieval by incubating the deparaffinized tissue sections in an EDTA buffer, pH 8.0, for 45 minutes. Secretagogin was detected using an affinity purified rabbit polyclonal anti-human secretagogin, antibody. (at 0.15 μg/ml, Sigma-Aldrich, # HPA006641) and tyramide signal amplification-based fluorescence detection of the secretagogin antibody (using a horse radish-conjugated anti-rabbit secondary antibody and a Rhodamine detection kit, from Ventana Medical Systems, # 760–4311 and # 760–233). Detection of insulin and glucagon was achieved by mouse monoclonal anti-glucagon (at 12.4 μg/ml, Sigma-Aldrich, # G2654) and polyclonal guinea pig anti-insulin, (at 17.4 μg/ml, DAKO # A0564), respectively, which were co-incubated with the secretagogin antibody on the tissue sections. The bound insulin and glucagon antibodies were then detected with Alexa Fluor 647 goat anti-mouse IgG and Alexa Fluor 488 goat anti-guinea pig IgG antibodies (both at 10 μg/ml, from Life Technologies). Then the tissue sections were counter-stained with DAPI (Sigma # D9542, final concentration 1 μg/ml) and cover slipped. Systematic cross-wise omission of each of the primary antibodies from the staining process resulted in complete loss of IHF staining in the relevant filter channel.

The IHF assay was used to stain one pancreatic tissue section from each of the eight normal controls and eight T2D subjects. The stained tissue sections were scanned using a 20x objective on a 3DHistech Panoramic 250 FLASH II digital slide scanner for visualization of the fluorophores. Each slide was scanned over the entire tissue area, using individual filter cubes optimized for viewing of the DAPI, Alexa Fluor 488, Rhodamine and Alexa Fluor 647 fluorophores. In addition, the tissues were simultaneously scanned through a fifth filter which exclusively detects tissue autofluorescence. Fixed exposure time and gain settings for imaging of the fluorophores and the tissue autofluorescence were selected to ensure that the images were not overexposed and that there was minimal cross-talk between the individual components of the IHF assay. This included evaluation and demonstration of absence of inadvertent bleed-over between the different filter channels (e.g. Rhodamine inadvertently generating signals through the Alexa Flour 488 or Cy5 filters) in single stained tissue sections scanned through all filters.

The image files were finally imported into software for quantitative image analysis (VIS, Visiopharm, Denmark) and processed for quantitative analysis of the deposited fluorophores. Briefly, the program was trained to specifically recognize and separate tissue areas that were labelled with each fluorophore above set thresholds. In addition, as means to avoid confounds from broad spectrum tissue autofluorescence the program was also trained to specifically recognize, and disregard, any tissue structures emitting autofluorescence above a set threshold. The program was finally programmed to measure the levels of IHF for secretagogin staining over tissue identified as stained positive for insulin or glucagon.

### EndoC-βH1 cell culture

EndoC-βH1 cells,a genetically engineered human pancreatic β-cell line exhibiting glucose-inducible insulin secretion [33], were cultured in Dulbecco's modified Eagle's medium (DMEM, Sigma Aldrich) low glucose (5.6 mM) containing 2% bovine serum albumin (BSA) fraction V, 50 μM 2-mercaptoethanol, 10 mM nicotinamide, 5.5 μg/mL transferrin, 6.7 ng/mL sodium selenite and penicillin (100 units/mL)/streptomycin (100 mg/mL). Cell culture plates were pre-coated with DMEM with 25mM glucose, containing fibronectin 2 μg/mL, ECM 1%, and penicillin (100 units/mL)/streptomycin (100 mg/mL). For the glucose-stimulated insulin release experiments, 50 000 cells were seeded in wells of coated 96-well plates. Cells were treated with 100 μL complete medium containing 5.5 mM glucose for 24 h then starved in Krebs-Ringer bicarbonate buffer containing 10mM Hepes, pH 7.4 (KRH), 0.2% BSA and 2.8 mM glucose for 1 h, and washed three times with KRH. The cells were then incubated in KRH with 2.8 mM or 16.7 mM glucose for 1h. Then cell culture medium was collected, cells were washed three times with PBS and immediately lysed in 100 μL RIPA lysis buffer (Sigma-Aldrich, #R0258).

### SiRNA transfection and treatment with stressing agents

EndoC-βH1 cells were transfected with FlexiTube pre-designed *SCGN* siRNA oligos (QIAGEN) using Lipofectamine RNAiMAX (Thermo Fisher Scientific) two days before treatment, according to the manufacturer's instructions. AllStars Negative Control siRNA (QIAGEN) was used as scrambled siRNA in all transfections. 50 000 EndoC-βH1 cells were seeded in wells of coated 96-well plates. Cells were treated with 100 μL complete medium containing 5.5 mM glucose and one of subsequent treatments; 1) DMSO (1:1000), 2) Thapsigargin (1 μM) in DMSO (1:1000), 3) Tunicamycin (10 μg/mL) in DMSO (1:1000), 4) Cytokine cocktail (IFN-γ (40 ng/mL), IL1-β (20 ng/mL), TNF-α (40 ng/mL)) in DMSO (1:1000) (all treatments, n = 4). After 24h, the medium was collected and cells lysed as described above. For normalization purpose, equal number of cells were seeded per well and the volume of medium and lysis buffer used was the same. The medium and protein extracts were kept at -80°C pending analysis.

The level of intracellular caspase 3/7 activity was assessed by Caspase-Glo assay systems (Promega, Madison, USA) according the manufacturer's instruction. Intracellular CCAAT-enhancer-binding protein homologous protein (CHOP) expression was assessed using western blotting analyzing 30 μl lysate/well (corresponding to 10μg total protein).

### Statistics

The results are presented as the mean±SD. Statistical analyses were performed using GraphPad Prism version 7.02. Group comparisons of clinical data was done using Mann-Whitney U-test. When multiple measurements existed for individual patients, the mean value for each patient was used. Correlations between secretagogin and other markers were assessed by the Spearman correlation coefficient (r). Group comparisons of *in vivo* and *in vitro* data were performed using unpaired Student’s t-test. Multiple comparisons in *in vitro* and IHF staining experiments were performed using one-way ANOVA with Dunnett’s multiple comparisons test or two-way ANOVA with Tukey’s correction for multiple testing. Values of *p*<0.05 was considered statistically significant.

## Results

### Abundant expression of secretagogin in human pancreatic islets

**Fig 1:**
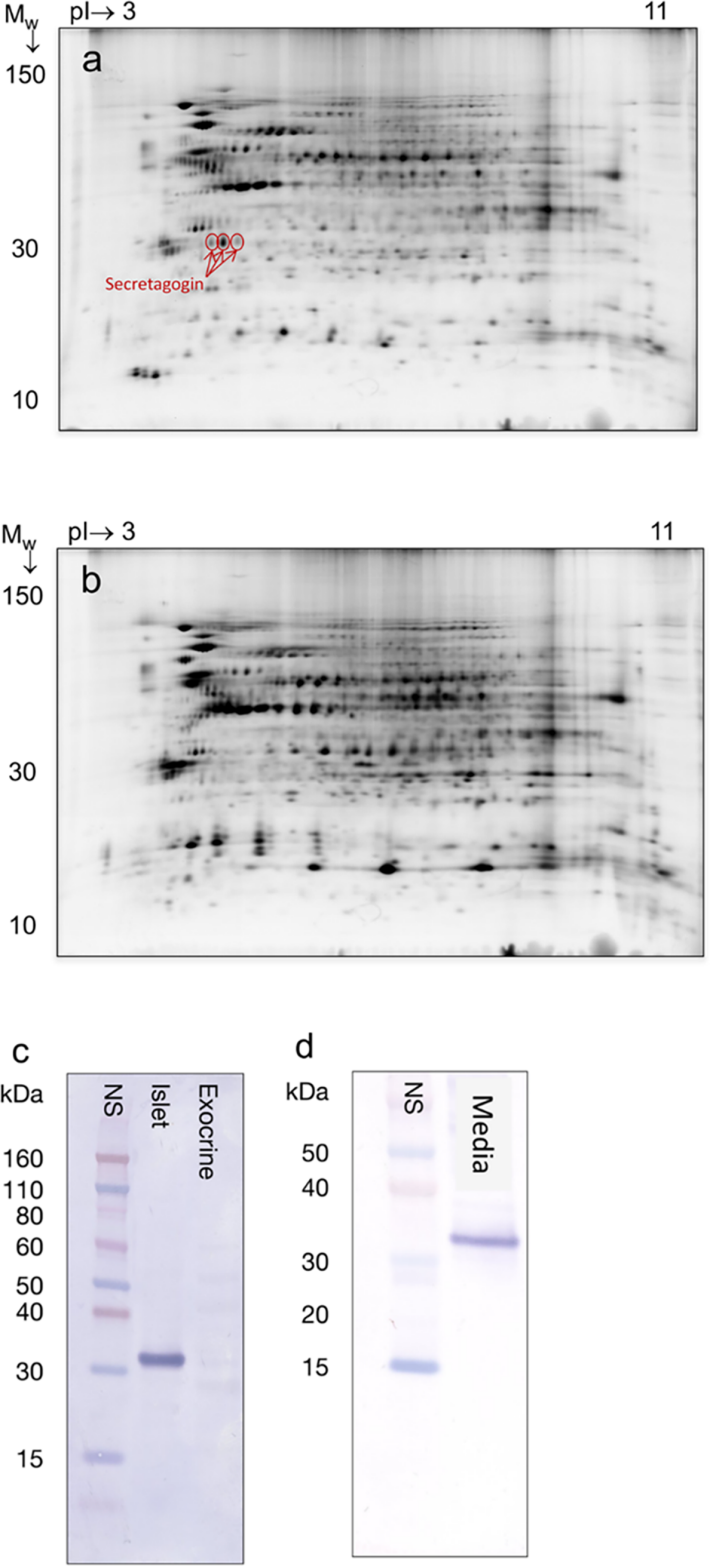
Abundant expression of secretagogin in human islets but not in exocrine tissue. Total protein extracts of human islets analyzed by two-dimensional difference in gel electrophoresis (DIGE). Protein spots identified as secretagogin by mass spectrometry (nano LC-FTICR MS/MS) are denoted in red. (b) Total protein extracts of exocrine pancreatic tissue analyzed by DIGE. (c) Western blot analysis of total extracts of human islets and exocrine pancreatic tissue and (d) conditioned media from human islets, immunostained with a polyclonal rabbit anti-secretagogin antibody.

Proteomic analysis of human islets was performed to identify proteins enriched in islets compared to exocrine pancreatic tissue. The 2D DIGE analysis allowed the simultaneous visualization in the same gel of islet proteins (Fig 1A) and exocrine proteins (Fig 1B). The protein spots were properly matched in the gel using a reference sample of equal concentrations of endocrine and exocrine human pancreatic tissue. The protein spots enriched more than two-fold in human islets compared with exocrine pancreas were selected for protein identification by MS. Among these proteins, secretagogin was abundantly expressed in the islets and identified in three distinct protein spots (Fig 1A and S1 Table).

A subsequent western blot analysis performed on extracts of human pancreatic islets and exocrine tissue, confirmed that secretagogin was expressed exclusively within the islets (Fig 1C). Released secretagogin was also detectable in conditioned media from cultured human islets by western blot (Fig 1D). Furthermore, the average level of secretagogin in media of islets from seven different donors was 13±4 pg/islet equivalent (IEQ), determined using ELISA.

### Elevated level of secretagogin in plasma from T2D patients

**Fig 2:**
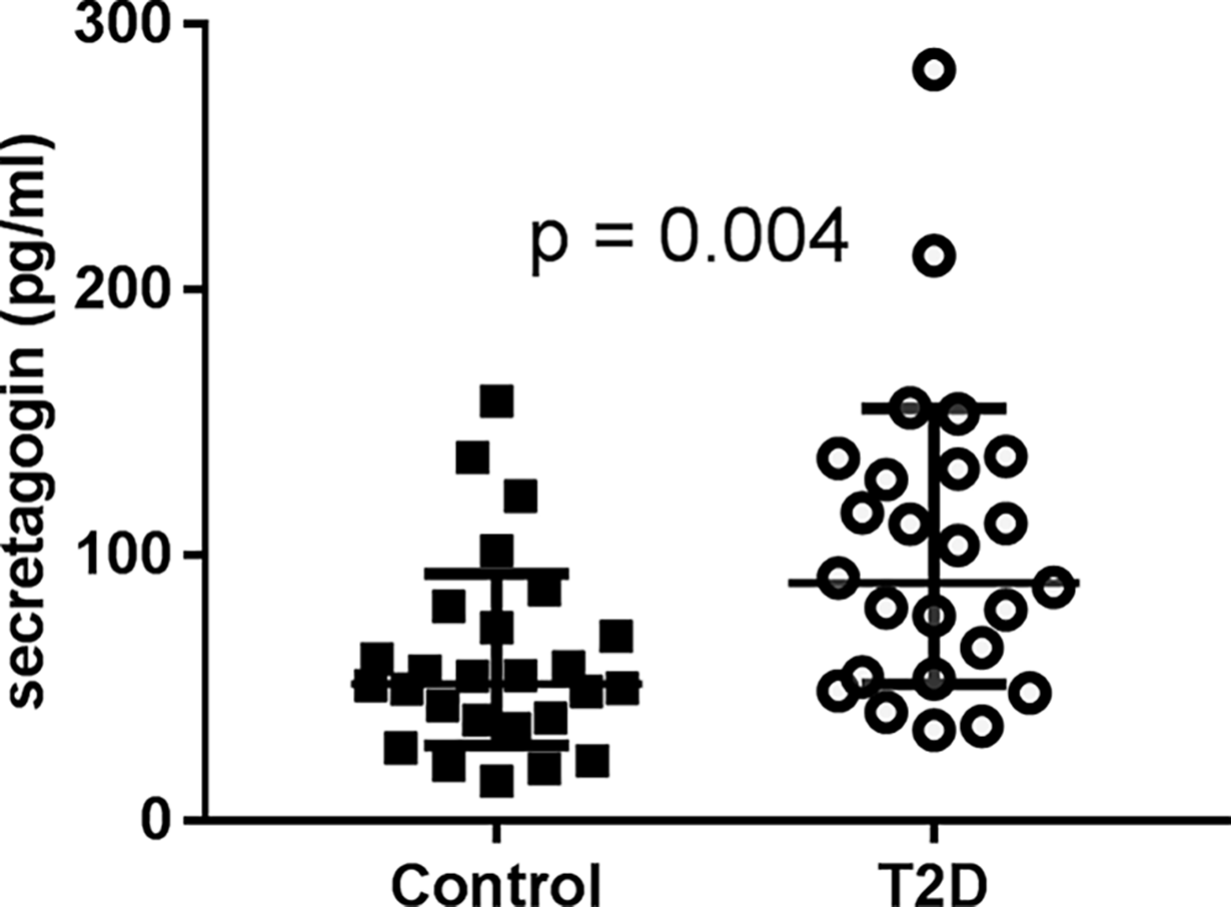
Plasma levels of secretagogin were increased in T2D patients. Secretagogin levels in plasma were significantly increased in T2D patients (n = 26) compared with age, BMI and gender matched healthy controls (n = 26) (p = 0.004). Data presented as mean±SD and statistical differences were calculated using Mann Whitney’s U test.

The potential of secretagogin as a serological biomarker was investigated by analyzing plasma levels of secretagogin in a clinical cohort of T2D and healthy control individuals (Table 1). The T2D patients of this cohort was well controlled, with mean HbA_1c_ levels of 6.8% ±1.0% and metformin was their only antidiabetic medication [30]. The plasma levels of secretagogin were significantly elevated in the T2D patients, when compared with non-diabetic control subjects (Fig 2).

One T2D subject was excluded from the data reported for the secretagogin plasma levels, since the level of that individual was about 50-fold higher than mean cohort levels and statistically defined as an outlier.

The subject characteristics and glycemic biomarkers and their correlation with plasma secretagogin levels were calculated and presented in Table 1. There was no association between plasma levels of insulin or glucagon with the secretagogin level. The most striking negative correlations were observed between secretagogin level and insulinogenic index (IGI) as well as HOMA2%B [34], respectively (Table 1). In contrast, secretagogin level had a positive correlation with fasting plasma glucose, fasting serum C-peptide and HbA_1c_, respectively.

### Increased plasma level of human secretagogin in mice with failing transplanted human islets

**Fig 3:**
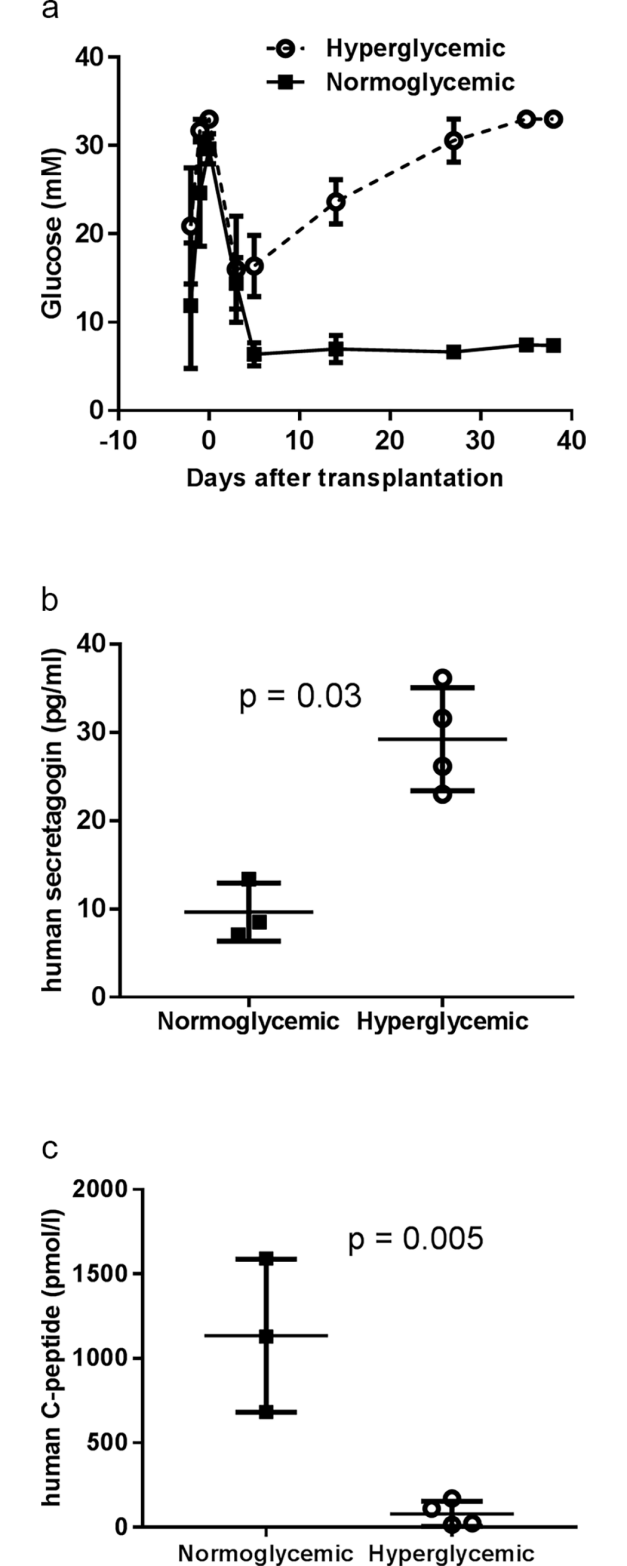
Plasma level of human secretagogin was increased as a result of human islet transplant failure in mice. (a) Plasma glucose levels in human islet-transplanted nu/nu mice. Mice (n = 7) were injected with alloxan to ablate the mouse beta cells, at day -3, followed by transplantation with human islets (1000 IEQ) under the kidney capsule at day 0. Plasma glucose level was monitored over 40 days. (b) Human secretagogin level (c) and human C-peptide level were measured in mouse plasma of both normoglycemic and hyperglycemic mice 40 days after human islet transplantation. Data are presented as mean±SD and statistical differences were calculated using Student's unpaired t-test.

To study the release of secretagogin from primary human islets *in vivo*, a human islet transplantation model was used. Seven hyperglycemic (32±0.7 mM glucose) nu/nu mice, with ablated endogenous islets, were transplanted with human islets under the kidney capsule, which resulted in a significant reduction in the plasma glucose level (13±2.4 mM) (Fig 3A). Three of the mice regained and remained normoglycemic (6.8±0.6 mM) during the entire observation period of 40 days. In contrast, the engraftment of the islets failed in four of the mice and they became increasingly hyperglycemic. In four weeks after the transplantation the plasma glucose level reached 30 mM again, in this group. Dividing the human islet transplanted mice into two groups, allowed comparison of secretagogin release from human islets *in vivo* under normoglycemic and diabetic conditions. The plasma level of human secretagogin was found to be increased in the diabetic mice compared with the normoglycemic mice (Fig 3B), while human C-peptide levels were decreased (Fig 3C). The results indicated that islet failure and/or the hyperglycemic condition resulted in increased secretagogin release from the islets as a possible measure of beta cell stress.

### Increased expression of secretagogin in β-cells from T2D pancreatic tissue sections

**Fig 4:**
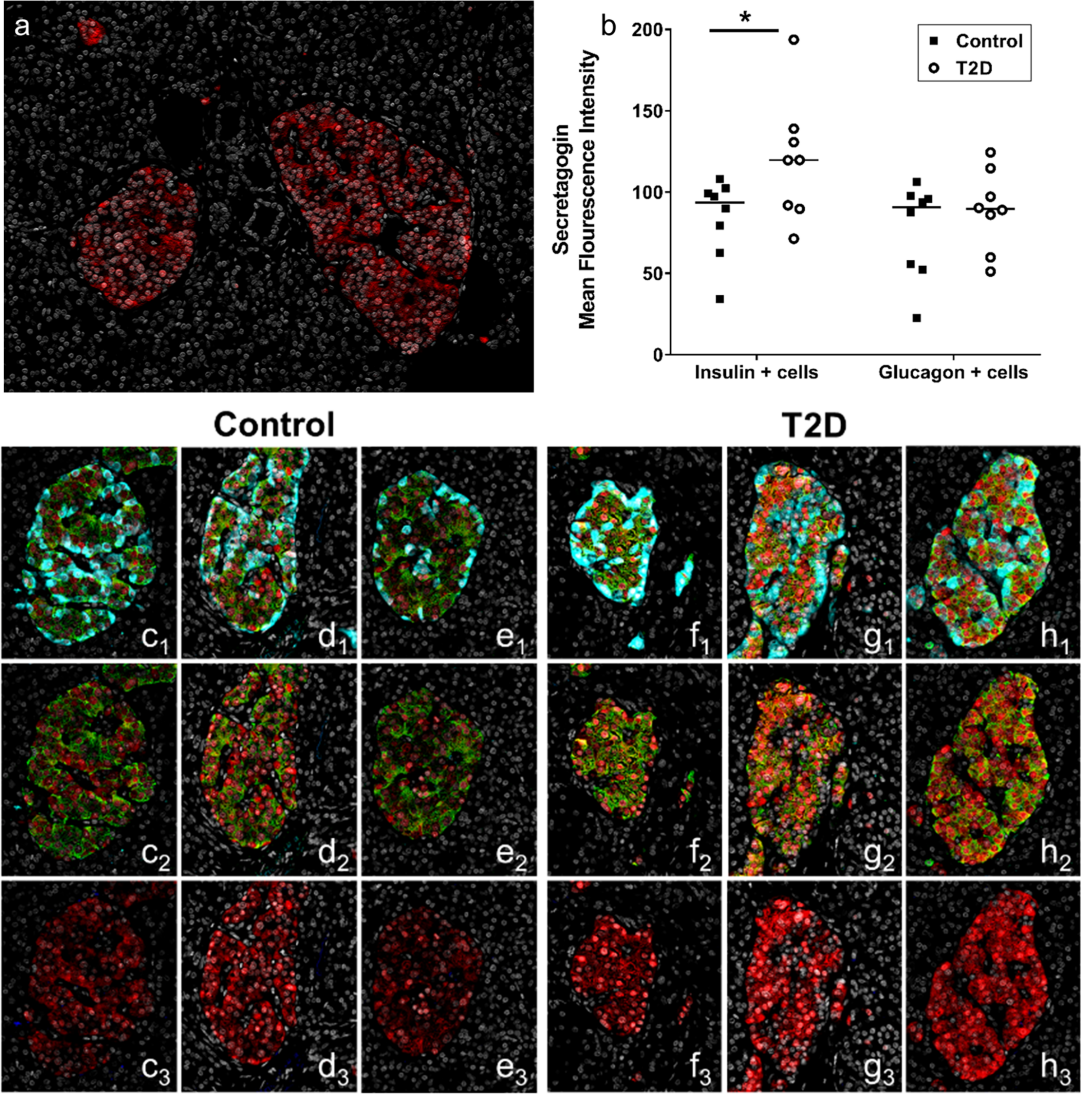
Increased secretagogin expression in beta cells from pancreatic tissue of T2D patients. (a) Secretagogin expression in islet cells of human pancreatic tissue sections were detected by immunohistofluorescence, using anti-secretagogin antibody (showed in red). Sections were counterstained with DAPI in nucleus (pseudo-colored in white). (b) Quantitative analysis of immunohistofluorescence as measured by mean fluorescence intensity of secretagogin staining in all insulin and glucagon expressing cells of human pancreatic tissue sections. Eight T2D individuals and eight control subjects, matched for age, BMI and gender, were used. Horizontal bars indicate group median. Group comparisons were performed using unpaired t-test. (c_1_, d_1_, e_1,_ f_1_, g_1_, h_1_) The upper row shows representative pictures of human islets from three control individuals (c_1_, d_1_, e_1_) and three T2D individuals (f_1_, g_1_, h_1_) (selected to reflect the group median staining intensities). Triple immunohistofluorescence staining was performed for secretagogin (showed in red), insulin (showed in green) and glucagon (showed in turquoise). (c_2_, d_2_, e_2,_ f_2_, g_2_, h_2_) The middle row shows secretagogin and insulin co-distribution (showed as yellow and orange tints). (c_3_, d_3_, e_3,_ f_3_, g_3_, h_3_) The lower row shows secretagogin immunostaining together with DAPI counterstaining (showed in white).

Using immunohistofluorescence (IHF) analysis the distribution of secretagogin within the human pancreas was characterized. This analysis confirmed an exclusive secretagogin immunoreactivity within the islet cells (Fig 4A). Furthermore, high resolution imaging demonstrated the presence of secretagogin in cytoplasm as well as nuclear compartment of both insulin and glucagon positive cells (S1 Fig). In addition, secretagogin staining was also observed in other islet cells not co-stained for either insulin or glucagon (S1 Fig).

Next, a quantitative analysis of the secretagogin staining intensity in insulin and glucagon-positive cells was performed in pancreatic tissue sections from T2D (n = 8) and non-diabetic controls (n = 8) (Table 2). It revealed a significantly enhanced secretagogin staining in insulin-positive, beta cells in T2D islets as compared with the non-diabetic controls (Fig 4B). In contrast, no significant difference in secretagogin staining intensity was observed in the glucagon-positive cells (Fig 4B). Representative pictures of IHF staining of human islets from three control individuals (Fig 4C_1_–4C_3_, 4D_1_–4D_3_ and 4E_1_–4E_3_) and three T2D individuals (Fig 4F_1_–4F_3_, 4G_1_–4G_3_ and 4H_1_–4H_3_) are shown.

### Secretagogin was not released in parallel with insulin from EndoC-βH1 cells

**Fig 5:**
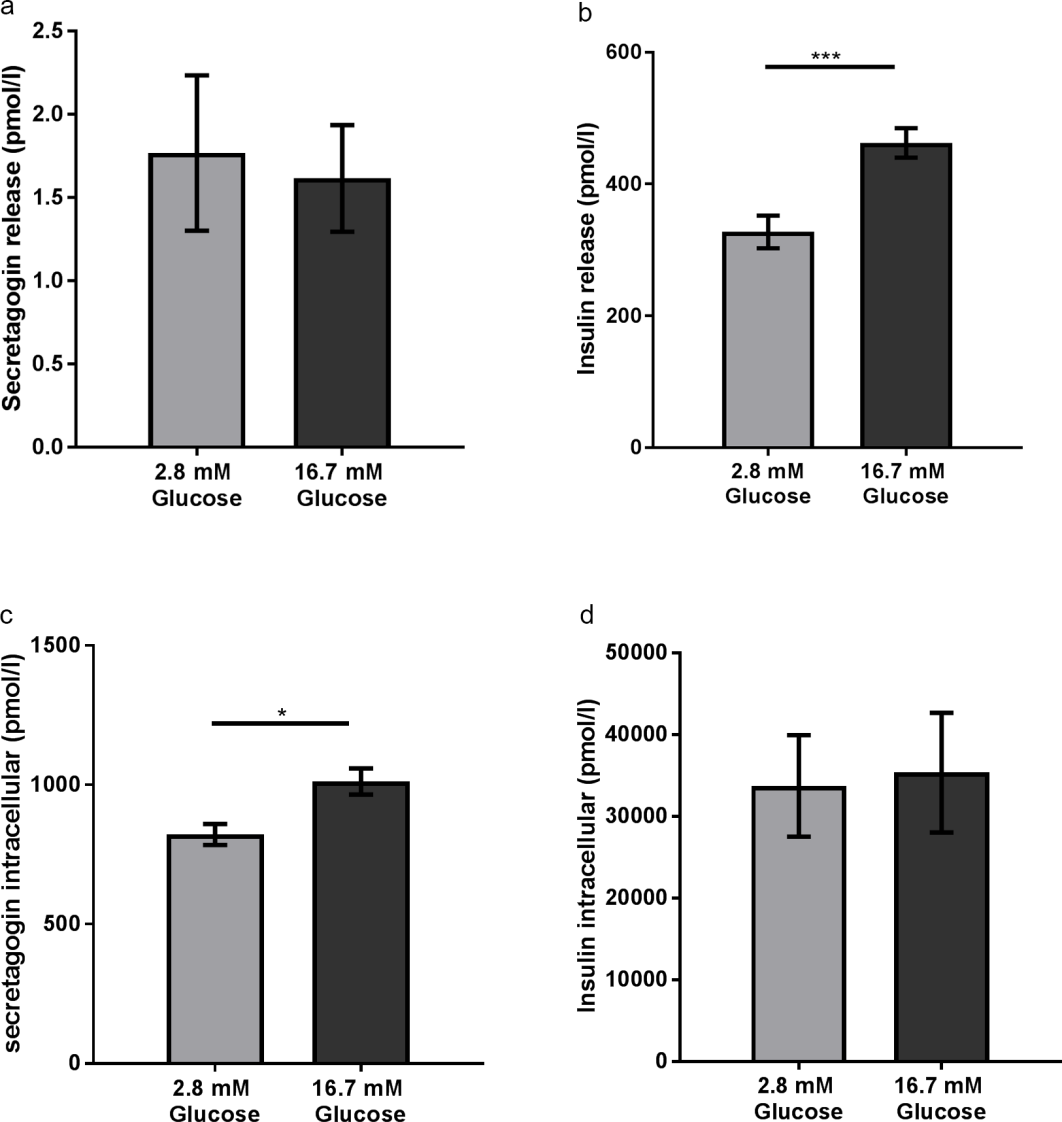
Secretagogin release was unaltered at different glucose concentrations. (a) Levels of secretagogin and (b) insulin released into cell media during 1 h incubation of EndoC-βH1 cells at 2.8 mM or 16.7 mM glucose level. (c) Intracellular levels of secretagogin and (d) insulin in cell lysates after 1 h treatment at low and high glucose concentration. Data are presented as mean ± SD. Statistical differences were calculated using Student’s unpaired t-test, n = 4. * *p* <0.05; *** *p* <0.0005.

To explore if secretagogin was secreted in parallel with insulin and/or released in response to hyperglycemic conditions, human EndoC-βH1 beta cells were treated with 2.8 mM or 16.7 mM glucose for 1h. Both secretagogin and insulin release as well as intracellular levels were measured. It showed that secretagogin release was unaltered at different glucose levels (Fig 5A), while there was a significant increase in insulin secretion at high glucose concentration (16.7 mM) compared with low concentration (2.8 mM) (Fig 5B). The intracellular level of secretagogin was slightly increased in EndoC cells by high glucose (Fig 5C), while the intracellular level of insulin was unchanged (Fig 5D). In all conditions, secretagogin was both released and expressed at substantially lower molar ratio (~200-fold lower release and ~40-fold lower intracellular expression) compared to insulin

### Treatment of EndoC-βH1 cells with stress-inducing agents increased the secretagogin release

**Fig 6:**
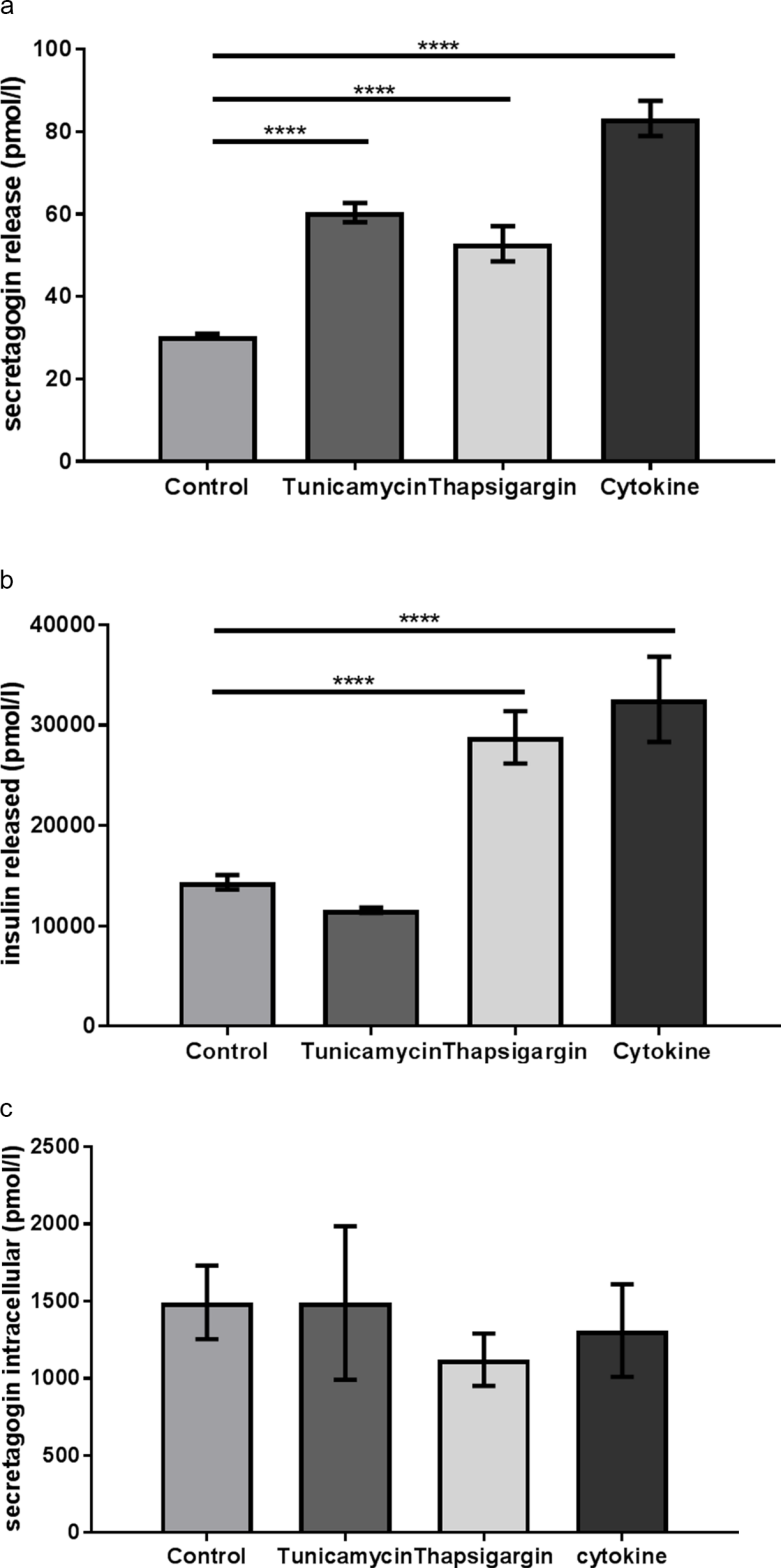
Secretagogin was released from human beta cells after induction of cellular stress. (a) Secretagogin release, (b) insulin secretion and (c) intracellular secretagogin level of EndoC-βH1 cells treated for 24h with stress inducers, either tunicamycin (10 μg/ml), thapsigargin (1 μM) or cytokine cocktail (IFN-γ (40 ng/ml), IL1-β (20 ng/ml), TNF-α (40 ng/ml)). All inducers were dissolved in DMSO (1:1000) and control cells were incubated in DMSO (1:1000). Data are presented as mean±SD, statistical differences were calculated using one-way ANOVA analysis, n = 4. **** *p*<0.0001.

To further explore what triggered the release of secretagogin, functional *in vitro* studies of ER and inflammatory stress were performed using human EndoC-βH1 cells. The secretagogin release from EndoC cells was significantly increased in response to 24 h treatment with all stressors, including tunicamycin, thapsigargin, and cytokine cocktail, compared with control cells (Fig 6A). The highest secretagogin release was triggered by cytokine cocktail, mimicking an inflammatory stress response (Fig 6A).

The insulin secretion was also measured in order to detect potential co-release of secretagogin. This showed that insulin and secretagogin were not released in parallel since the pattern of release was different for the two proteins (Fig 6A and 6B). Thapsigargin and cytokine treatments significantly increased the release of both insulin and secretagogin from the cells (Fig 6B). However, tunicamycin incubation triggered only secretagogin release with no parallel increase of insulin secretion when compared with control cells. These results indicate that secretagogin and insulin can be released independently of each other.

In terms of intracellular secretagogin levels, none of the cellular stress inducing treatments resulted in significant changes (Fig 6C). It suggests that the intracellular secretagogin does not necessarily drive its increased release into the cell media.

### Intracellular secretagogin protected EndoC-βH1 cells from ER stress-induced apoptosis

To confirm that the stress inducers (tunicamycin, thapsigargin and cytokine cocktail) lead to apoptosis, the levels of caspase 3/7 activity was assessed in parallel to the secretagogin and insulin release. Furthermore, the causative effect of secretagogin on EndoC-βH1 apoptosis was determined by siRNA silencing of secretagogin.

For effective secretagogin silencing four different secretagogin siRNA oligos (siSCGN), #4 to #7, and a pool of the oligos (with lower dose of each oligo, were tested (S2 Fig)). The pooled siRNA-oligos were most effective in silencing of the intracellular secretagogin mRNA levels and subsequently used in the experiments. The pooled siSCGN oligos silenced secretagogin mRNA expression by 89% and 93% (normalized to housekeeping gene HPRT1) compared with scrambled siRNA-treated control cells after 48 h and 72 h respectively. On protein level this corresponded to a reduction of intracellular secretagogin expression by 54% as measured by ELISA in lysates of silenced EndoC cells (p<0.0001) compared with non-silenced cells. This indicated that the secretagogin protein was fairly stable since a substantial suppression of the mRNA expression only resulted in reduction of protein expression to about 50%.

**Fig 7:**
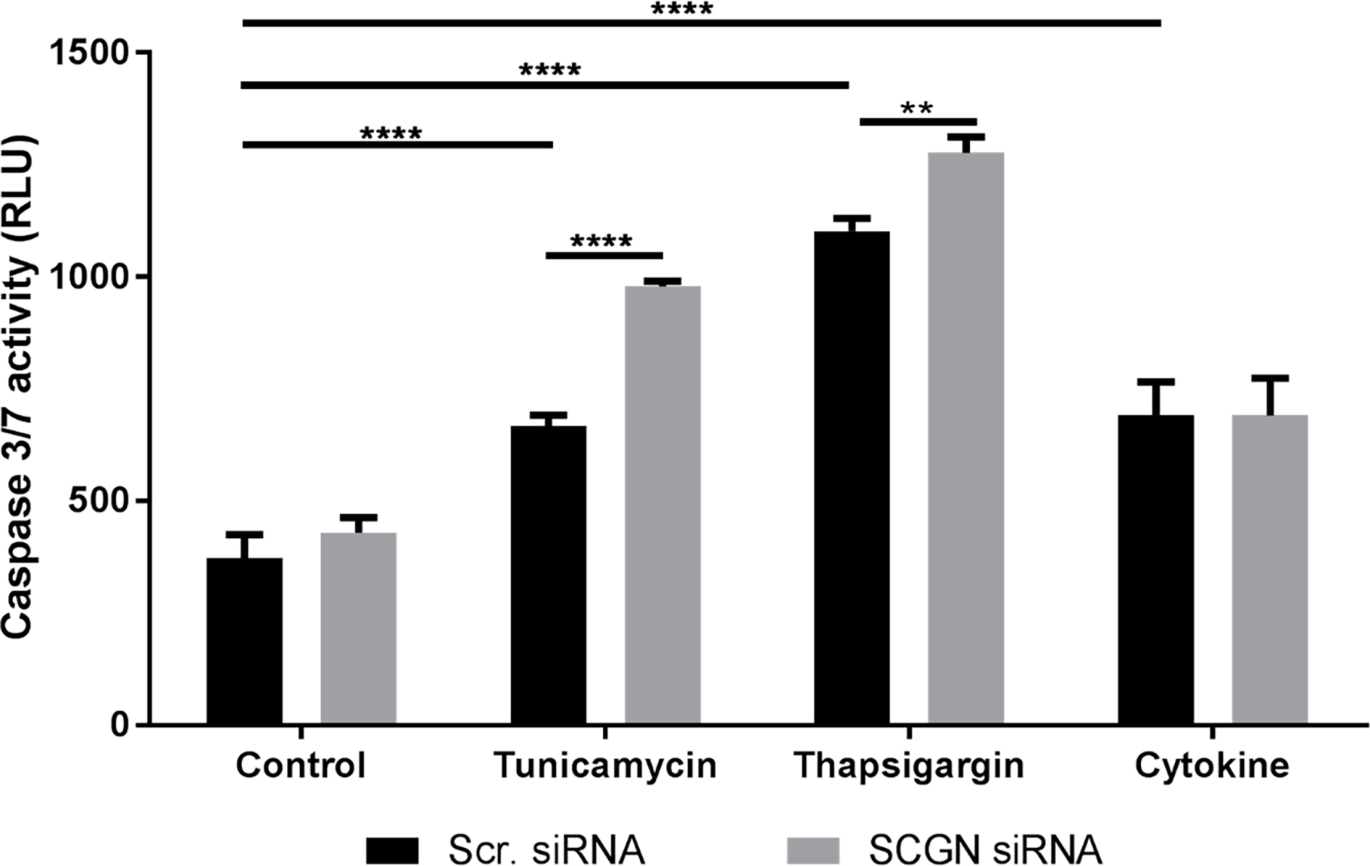
Silencing of secretagogin in EndoC cells increased ER stress-induced apoptosis. Caspase 3/7 activity in EndoC-βH1 cells pretreated (48h) with scrambled control or secretagogin siRNA followed by stress induction by either tunicamycin, thapsigargin or cytokine cocktail (IFN-γ, IL1-β, TNF-α) for 24h. All substances were dissolved in DMSO (1:1000) and control cells were incubated in DMSO (1:1000). Scr. siRNA = Cells treated with scrambled siRNA. siSCGN = Secretagogin knock down by siRNA. Data are presented as mean ± SD and statistical differences were calculated using two-way ANOVA analysis, n = 4. ** *p* = 0.003; **** *p*< 0.0001.

All tested stress-inducing agents resulted in significantly increased apoptosis in human EndoC-βH1 cells (Fig 7). Despite only 50% decrease in protein level, silencing of secretagogin in EndoC beta cells resulted in further enhanced caspase 3/7 activity after 24h treatments with the ER stressor tunicamycin or thapsigargin (Fig 7). Moreover, the activation of unfolded protein response (UPR) by ER stressors was assessed using immunoblotting of CHOP in protein lysates. Only tunicamycin and thapsigargin treatments but not cytokine cocktail induced CHOP protein expression (S3 Fig).

## Discussion

In this study we explored the role of increased release of secretagogin as a potential biomarker of human islet stress and revealed that T2D patients had elevated plasma level of secretagogin. Secretagogin was identified as a candidate biomarker present in human islets through a proteomic analysis. It showed that secretagogin was highly abundant in the islets but not present in the exocrine pancreas, which was further confirmed with immunoblotting. We also found that secretagogin was released into media from primary human islets, which is in line with a previous study from rat insulinoma Rin-F cells [19]. These findings raised an intriguing question whether secretagogin can be used as a soluble biomarker of islet cell stress, given its published association with ER stress in beta cell [28]. To answer this question, we assessed secretagogin levels in plasma samples from healthy and T2D subjects. The T2D individuals in this study had a well-controlled diabetes disease, with metformin as their only antidiabetic medication. In this study we observed that:

1. The secretagogin plasma level was elevated in T2D patients compared with matched healthy controls.
2. The secretagogin plasma level was inversely associated with HOMA2%B (an estimate of steady state beta cell function [34]) and insulinogenic index (index of beta cell function [35]), supporting its potential as a marker of beta cell or islet stress status.
3. The secretagogin plasma level was not associated with insulin or glucagon levels, indicating a different mechanism of release or degradation/removal from plasma between secretagogin and the beta and alpha cell specific hormones.
4. There was a nominally significant positive correlation between secretagogin and fasting glucose, fasting C-peptide, and HbA_1C_ levels, suggesting that a disturbed glucose homeostasis may be related to increased secretagogin plasma levels.

As a potential biomarker of islet stress status in T2D patients secretagogin may have some limitations. For instance, secretagogin has been reported to be highly expressed in some endocrine form of cancers [26,36–38]. Moreover, secretagogin was identified as a potential biomarker of cerebral ischemia damage or stroke [21,39]. This may result in altered plasma level of secretagogin in these disorders as well. In a study by Montaner *et* al. [39] the secretagogin plasma level was quantified in 915 stroke patients and 90 patients with stroke mimicking conditions. Their study indicated that stroke patients had lower levels of secretagogin compared with the mimicking conditions. Then they assessed the effect of different risk factors in this stroke cohort, including T2D, on the plasma concentration of secretagogin. Without knowing the source tissues of secretagogin, they found that the T2D population (n = 260) had elevated plasma levels of secretagogin compared with non-T2D individuals (n = 745) (*p*-value = 0.02) in their cohort [39]. The study by Montaner and colleagues supports the finding of increased secretagogin levels in our well controlled early T2D cohort. It also suggests that plasma secretagogin as a biomarker is distinguishable between T2D and cerebral ischemia. Given our findings of negative correlation with clinical estimates of beta cell function, we hypothesize that islets predominantly contribute to the increase of secretagogin level in T2D plasma, although islets are not the only source tissue of secretagogin release [17,22].

To evaluate the extent of secretagogin release exclusively from human islets *in vivo*, we adopted a mouse model of human islet transplantation, in which successful transplantation resulted in normoglycemia while transplant failure lead to islet dysfunction and hyperglycemia in the mice. Using this model, we could confirm that levels of human specific secretagogin were significantly increased in the plasma of mice with hyperglycemia, which was likely caused by beta cell failure. This finding supports the concept that secretagogin release from the islets was sufficient to create quantifiable levels in the plasma as part of islet failure (i.e. increased islet stress).

The IHF analysis in our study demonstrated that secretagogin was expressed in all islet cells but not in the exocrine cells. This is in line with a recent study using single cell RNA-seq analysis of human pancreatic islets showing high mRNA expression of secretagogin in all endocrine islet cells, with a trend of higher expression in beta cells [40]. Furthermore, increased intracellular levels of secretagogin were found specifically in the beta cells of the T2D subjects compared with controls. In contrast to our findings, Malenzcyk *et* al. [28], recently reported that the mRNA level of secretagogin was significantly reduced in isolated islets from T2D donors compared with healthy controls. The discrepancy between these findings may be explained by that secretagogin mRNA level may not correspond to the immunohistochemically detected protein level, as indicated in a previous study [20]. An alternative explanation may be that the isolation of culture of islets for one to nine days prior to mRNA analysis may affect the mRNA expression level. Nevertheless, our IHF analysis should be interpreted with caution, as only one tissue slide per individual was used due to the limitation of material and a fairly large variation of secretagogin levels was observed between T2D individuals. Additionally, the quantitative analyses of the IHF-stained tissue sections were performed on images acquired with a fluorescence scanning microscope. The chosen approach for imaging provided important benefits (e.g. generating quantitative data from all endocrine cells within each tissue in a robust and non-biased way and rapid acquisition of evenly illuminated images), this scanning microscope offers a lower resolution in the Z plane as compared to confocal microscopes. This increases the risk that a component of our dual staining data may actually be derived from IHF staining of different but locally overlapping cells. The potential impact from such an overlap was limited by using 4 μm thin tissue sections for analysis of the endocrine cells (roughly sized 8–15 μm).

To investigate whether hyperglycemia and/or induced islet stress resulted in increased secretagogin release the pattern of its release was assessed *in vitro* in response to either high glucose levels or stress induction. As pharmacological inducers of ER stress, tunicamycin (an inhibitor of N-linked protein glycosylation) and thapsigargin (an inhibitor of sarcoplasmic/endoplasmic reticulum Ca^2+^ ATPase [SERCAs]) were used. The pro-inflammatory stress mechanism was mimicked by treatment using the combination of IFN-γ, IL1-β and TNF-α as a cytokine cocktail. These *in vitro* studies showed that:

1. Secretagogin was released in response to both ER stress and cytokine stimuli in human beta cells. The siRNA knockdown of intracellular secretagogin increased apoptosis of EndoC cells only in response to ER stress inducers.
2. Secretagogin was not released in parallel with insulin and not in response to hyperglycemic conditions. The intracellular level of secretagogin was slightly upregulated by high glucose level in EndoC cells.

Our findings that intracellular secretagogin protected beta cells from apoptosis is supported by recent findings in cancer cells by Bai and colleagues [26] which shows that overexpression of secretagogin inhibited apoptosis. Interestingly, in our study the anti-apoptotic effects of secretagogin were only seen in cells that were subjected to pharmacological ER stressors but not to the cytokine cocktail. Secretagogin has been suggested to be a Ca^2+^ sensor and not a buffering protein [27]. However, it was recently proposed that depending on availability of Ca^2+^ and redox state secretagogin may have different characteristics [41]. In the cytosol, secretagogin may then function as a Ca^2+^ sensor, while in an oxidizing milieu (such as in ER and under oxidative stress conditions) secretagogin may act as a Ca^2+^ buffer [41]. Fluctuations in the levels of Ca^2+^ in the ER can severely impact protein folding capacity, causing UPR and triggering cell death [42]. Thus, in ER, secretagogin may reduce Ca^2+^ fluctuations and promote survival. These results are also in line with a recent finding that intracellular secretagogin promotes pancreatic beta cell survival and decreases ER stress by stabilizing deubiquitinating proteins [28].

Secretagogin has previously been suggested to be involved in beta cell homeostasis by protecting against IL-1β-mediated beta cell destruction in diabetes-prone biobreeding rat islets [43]. These results are in contrast with our study, which indicated that cytokines did not change the apoptotic susceptibility of secretagogin silenced human beta cells. However, another study showed that intracellular secretagogin expression was not affected by cytokines in rat insulinoma cells and neither in islets from Wistar Furth rats[25]. The discrepancy between different studies highlights that use of different strains and model systems may give conflicting results.

Previous studies based on rodent models, or rodent beta cell lines [23,24,27] have shown that intracellular secretagogin modulates and facilitates the exocytosis of insulin through interaction with cytoskeletal proteins [24,44], soluble N-ethylmalemide-sensitive-factor attachment receptor (SNARE) components [45,46] and cargo proteins [47]. The tight association of secretagogin with the exocytosis machinery may postulate a release during insulin secretion. However, in this study we show that secretagogin release is not in parallel to glucose stimulated insulin secretion, which has also been shown in a study of secretagogin release from rodent beta cells [19].

The fact that secretagogin release was not fluctuating in relation to insulin release is an important feature of a novel biomarker. It could enable secretagogin to reflect a certain disease condition that cannot be captured by insulin, C-peptide or proinsulin levels. A suggestion may be that secretagogin may function as a stress responsive endocrine or paracrine factor, released in response to increased cell stress. But further studies on effects of released secretagogin on surrounding cells are needed to test this hypothesis.

In conclusion, our findings suggest that released secretagogin is a candidate plasma biomarker of islet cell stress with independent value compared to insulin. To further validate secretagogin as a biomarker for islet stress, studies in larger cohorts are warranted.

## Supporting information

S1 Fig Secretagogin was expressed in cytoplasm and nuclear compartment of both insulin and glucagon positive cells. High resolution immunohistofluorescence staining for secretagogin (showed in red), insulin (showed in green) and glucagon (showed in turquoise), and nucleus counterstained with DAPI (pseudo-colored in white). In the left image secretagogin is visualized together with the nuclear counterstaining. In the middle image, the insulin staining is added to the secretagogin and nuclear staining. In the right-hand image, secretagogin, insulin and nuclear staining is visualized together with the staining for glucagon.
(TIF)

S2 Fig A pool of silencing oligos gave robust knock down of secretagogin mRNA levels in EndoC cells. Four pre-designed (FlexiTube, QIAGEN) silencing RNA oligos of secretagogin (siSCGN) as well as a pool of #4-#6 were assessed for their effect on mRNA silencing of secretagogin expression in EndoC cells as compared with the expression of a scrambled siRNA (Scr siRNA) as negative control.
(TIF)

S3 Fig CHOP expression was induced in EndoC cells treated with thapsigargin and tunicamycin. Intracellular CCAAT-enhancer-binding protein homologous protein (CHOP) expression was assessed using western blotting, analyzing 10μg total protein per well of EndoC cells treated with stress induction by either tunicamycin, thapsigargin or cytokine cocktail (IFN-γ, IL1-β, TNF-α) for 24h. All substances were dissolved in DMSO (1:1000) and control cells were incubated in DMSO (1:1000).
(TIF)

S1 Table Identification of secretagogin from 2D gel analysis by mass spectrometry. (DOCX)

S1 Material and Methods Proteomics analysis. (DOCX)
