# Supplementary figures and images for: Secretagogin is increased in plasma from type 2 diabetes patients and potentially reflects stress and islet dysfunction
Source: PLoS One. 2018 Apr 27;13(4):e0196601. doi: 10.1371/journal.pone.0196601 (PMC5922551; doi:10.1371/journal.pone.0196601)

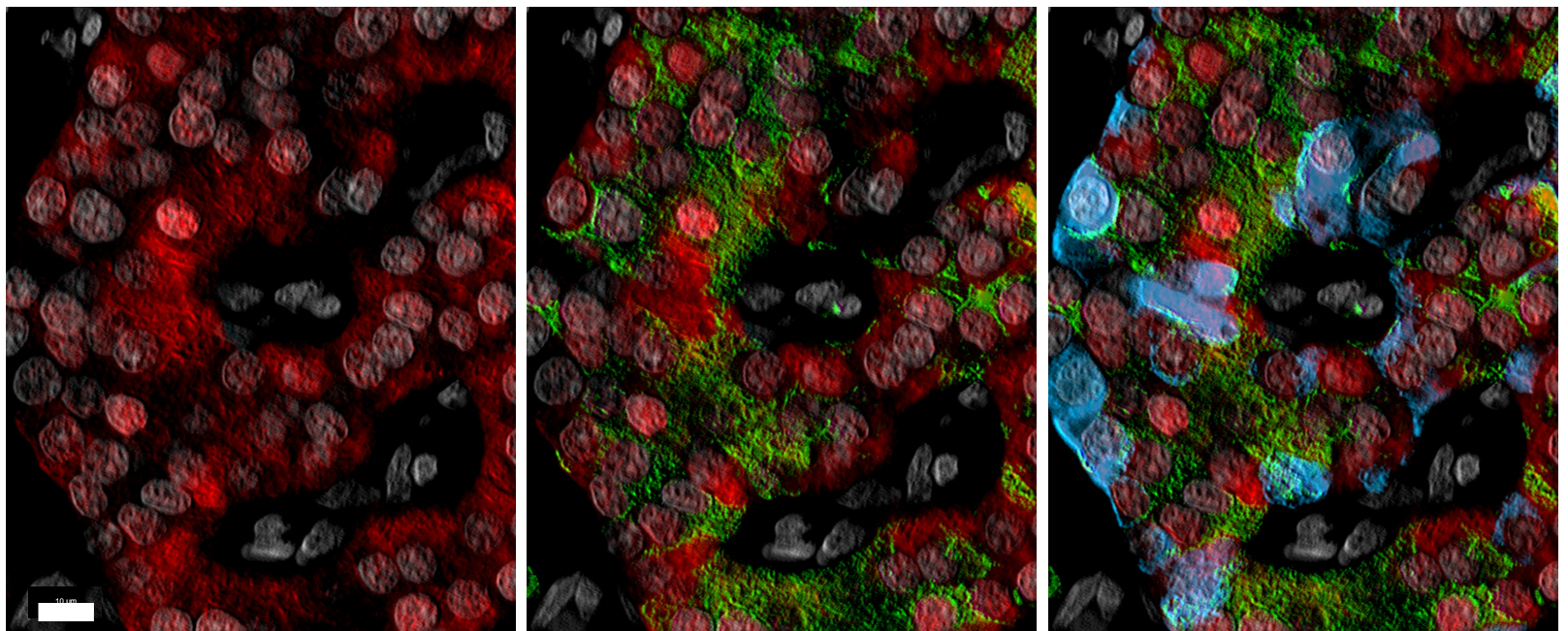

Supplement: S1 Fig — High resolution immunohistofluorescence staining for secretagogin (showed in red), insulin (showed in green) and glucagon (showed in turquoise), and nucleus counterstained with DAPI (pseudo-colored in white). In the left image secretagogin is visualized together with the nuclear counterstaining. In the middle image, the insulin staining is added to the secretagogin and nuclear staining. In the right-hand image, secretagogin, insulin and nuclear staining is visualized together with the staining for glucagon. (TIF) [file pone.0196601.s001.tif]

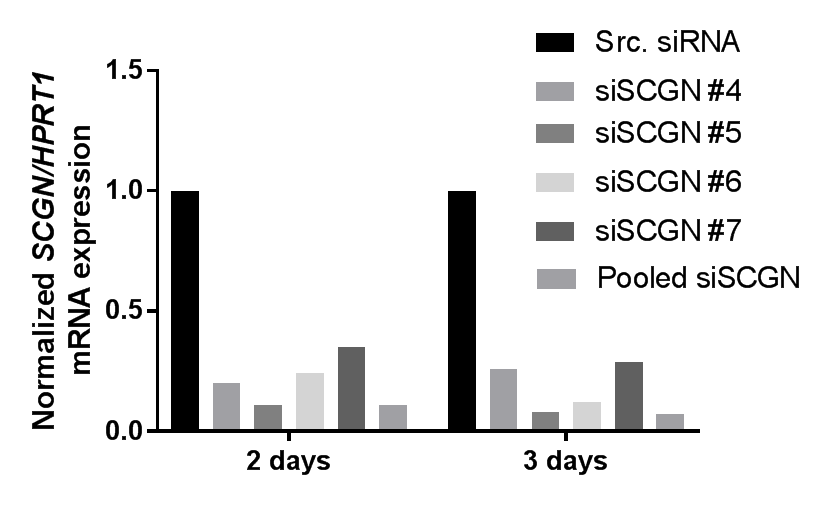

Supplement: S2 Fig — Four pre-designed (FlexiTube, QIAGEN) silencing RNA oligos of secretagogin (siSCGN) as well as a pool of #4-#6 were assessed for their effect on mRNA silencing of secretagogin expression in EndoC cells as compared with the expression of a scrambled siRNA (Scr siRNA) as negative control. (TIF) [file pone.0196601.s002.tif]

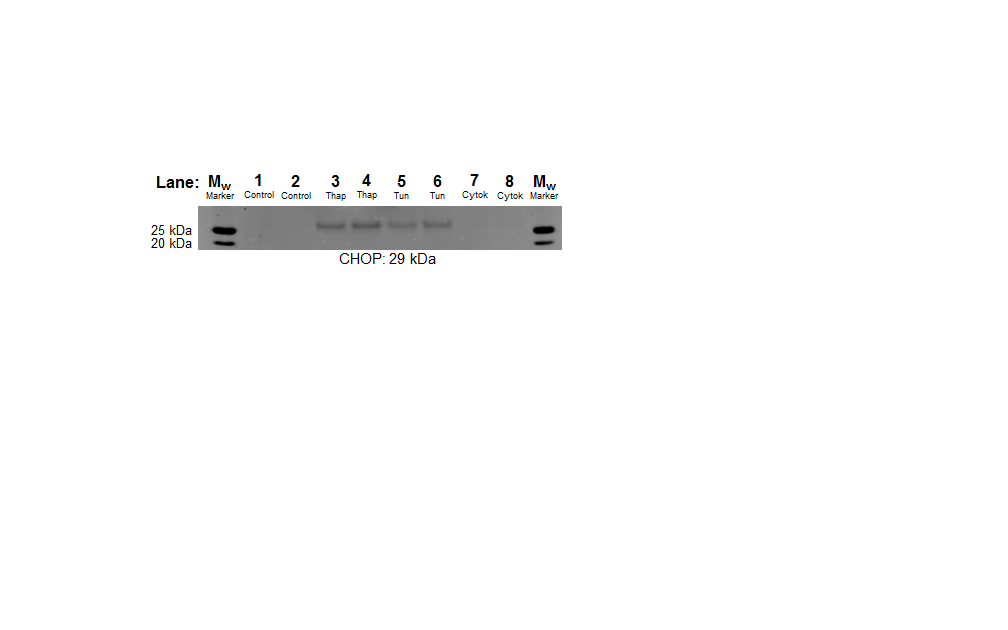

Supplement: S3 Fig — Intracellular CCAAT-enhancer-binding protein homologous protein (CHOP) expression was assessed using western blotting, analyzing 10μg total protein per well of EndoC cells treated with stress induction by either tunicamycin, thapsigargin or cytokine cocktail (IFN-γ, IL1-β, TNF-α) for 24h. All substances were dissolved in DMSO (1:1000) and control cells were incubated in DMSO (1:1000). (TIF) [file pone.0196601.s003.tif]
