# Supplementary material for: Secretagogin is increased in plasma from type 2 diabetes patients and potentially reflects stress and islet dysfunction
Source: PLoS One. 2018 Apr 27;13(4):e0196601. doi: 10.1371/journal.pone.0196601 (PMC5922551; doi:10.1371/journal.pone.0196601)
Supplement: S1 Table — (DOCX) [file pone.0196601.s004.docx]

**S1 Table. Identification of secretagogin from 2D gel analysis by mass spectrometry**

| **Spot no*** | **Accession (UniProtKB)** | **# AAs** | **MW [kDa]** | **calc. pI** | **Protein name** | **# PSMs** | **# Peptides** | **Peptide sequence** | **Score (mascot)** | **emPAI** |
| --- | --- | --- | --- | --- | --- | --- | --- | --- | --- | --- |
| 1 | O76038 | 276 | 32.0 | 5.41 | Secretagogin | 56 | 15 | R.DLFLHHK.K R.LDLNDLAR.I K.LGTDDTVMK.A R.DLFLHHKK.A K.IFAYYDVSK.T K.LEEYTGTMMK.I K.DGRLDLNDLAR.I K.QQFMTTQDASK.D K.TGALEGPEVDGFVK.D R.FDADEKGYIEEK.E K.VKQQFMTTQDASK.D K.LGTDDTVMKANLHK.V K.QQFMTTQDASKDGR.I R.KYDADSSGFISAAELR.N K.AISEAKLEEYTGTMMK.I | 701 | 5.59 |
| 2 | O76038 | 276 | 32.0 | 5.41 | Secretagogin | 143 | 18 | R.DLFLHHK.K R.LDLNDLAR.I K.LGTDDTVMK.A R.DLFLHHKK.A K.IFAYYDVSK.T K.LEEYTGTMMK.I K.DGRLDLNDLAR.I K.QQFMTTQDASK.D K.TGALEGPEVDGFVK.D R.FDADEKGYIEEK.E K.ELDAFFLHMLMK.L K.VKQQFMTTQDASK.D K.LGTDDTVMKANLHK.V K.YDADSSGFISAAELR.N K.QQFMTTQDASKDGR.I R.KYDADSSGFISAAELR.N K.AISEAKLEEYTGTMMK.I K.DMMELVQPSISGVDLDK.F | 972 | 11.81 |
| 3 | O76038 | 276 | 32.0 | 5.41 | Secretagogin | 48 | 13 | R.DLFLHHK.K R.LDLNDLAR.I K.LGTDDTVMK.A K.IFAYYDVSK.T K.LEEYTGTMMK.I K.DGRLDLNDLAR.I K.QQFMTTQDASK.D K.TGALEGPEVDGFVK.D R.FDADEKGYIEEK.E K.VKQQFMTTQDASK.D K.LGTDDTVMKANLHK.V K.QQFMTTQDASKDGR.I K.AISEAKLEEYTGTMMK.I | 611 | 4.89 |
| * denoted from left to right in the gel. # AA= number of amino acids, # PSM = number of peptide-spectrum matches | | | | | | | | | |  |
