# Supplementary material for: Secretagogin is increased in plasma from type 2 diabetes patients and potentially reflects stress and islet dysfunction
Source: PLoS One. 2018 Apr 27;13(4):e0196601. doi: 10.1371/journal.pone.0196601 (PMC5922551; doi:10.1371/journal.pone.0196601)
Supplement: S1 Material and Methods — (DOCX) [file pone.0196601.s005.docx]

**S1 Material and Methods**

**Proteomic analysis**

**Two-dimensional gel electrophoresis and in gel digestion**

Proteins were extracted from human islets and exocrine pancreas with 7M urea, 2M thiourea, 50 mM tris-HCL, 50mM SDS, 50 mM CHAPS, pH 8.0 and protease inhibitors (s-PEK kit,) followed by protein precipitation (ProteoExtract, Calbiochem, Merck Chemicals). Proteins were solubilized in phosphate buffered saline (PBS) and the total protein concentration was determined using bicinchoninic acid (BCA) protein assay kit (Pierce, Life Technologies). Total protein extracts (50 µg of each, labelled with 400 pmol CyDye) of exocrine pancreatic tissue (Cy3) and endocrine islets (Cy5) as well as an equimolecular reference extract of pooled exocrine and endocrine proteins (Cy2) were analyzed with differential in gel electrophoresis (DIGE, GE-healthcare). Proteins were resolved in 7M Urea, 2M Thiourea, 4% 3-((3-cholamidopropyl) dimethylammonio)-1-propanesulfonate (CHAPS), 1.2% DeStreak, 0.5 % IPG buffer and bromophenol blue and separated in the first dimension using immobilized pH gradient (IPG) strips (GE healthcare), 24 cm, pH 3-11 non-linear gradient. The IPG-strips were rehydrated in the protein sample for 12 h at 30 V followed by protein focusing for 100 000 Vh using the Ettan IPGphor (GE healthcare). Each strip was equilibrated with buffer (50 mM Tris-HCl pH 8.8, 6M urea, 30% glycerol, 2% SDS, 0,01% bromophenol blue) first containing 1% DTT, and then 2.5% iodoacetamide. The second dimension were run on an Ettan DALT II 1 mm polyacrylamide 8-20% Bis‐Tris gel with standard MOPS cathode buffer and acetic acid/diethanol amine anode buffer. After 2D electrophoresis, gels were scanned using the 2920 Master Imager (Amersham Bioscience, Uppsala, Sweden) using excitation/emission wavelengths specific for the different CyDyes. The protein spot pattern was analyzed (Progenesis SameSpots, Nonlinear Dynamics Ltd., UK) and proteins, at least two-fold increased in the islets of Langerhans compared with exocrine pancreatic tissue were cut, using a robotic system, from a preparative 2DE gel containing islet protein extract (200 µg protein) stained using SYPRO Ruby Protein Stain (Molecular-Probes, Eugene, Oregon, USA).

Selected 2D gel spots were subjected to in gel digestion, using 100 ng trypsin/spot (Promega, Madison, USA as previously described by Shevchenko et al. [[28](#_ENREF_28)]. Prior to mass spectrometry (MS) analysis, the peptides were reconstituted in 0.1% HCOOH.

**On-Line Nano-LC Separation**

Sample injections (2 µl) were made with an HTC-PAL auto sampler (CTC Analytics AG, Zwingen, Switzerland) equipped with a Cheminert valve (0.25 mm bore, C2V-1006DCTC, Valco Instruments Co, Schenkon, Switzerland), connected to an Agilent 1100 binary pump (Agilent Technologies, Palo Alto, CA, USA). The peptides were trapped on a precolumn (45 x 0.075 mm i.d.) and separated on a reversed phase column, 200 x 0.050 mm i.d. fused silica column packed in-house with 3 µm ReproSil-Pur C18-AQ porous (120Å) C18- bonded particles (Dr. Maisch GmbH, Ammerbuch, Germany). A 30 min gradient, 10-50% CH3CN in 0.2% COOH at 100 nl/min was used for separation of the peptides. For more details see [[29](#_ENREF_29)].

**Mass spectrometry**

The nanoflow LC-MS/MS were performed on a hybrid linear ion trap-FT-ICR mass spectrometer equipped with a 7T ICR magnet (LTQ-FT, Thermo Electron, Bremen, Germany) using capillary voltage and temperature of 42 V and 225°C, respectively, and a tube lens voltage of 120 V. The mass spectrometer was operated in data-dependent mode to automatically switch between MS and MS/MS acquisition. Survey mass spectra (from m/z 400- 1600) were acquired in the FT-ICR mass spectrometer with a resolving power of 60,000 at m/z 600 and a target value of 500 000. Automatic gain control (AGC) 32 was used for sending in an accurate number of ions to the ICR cell. The six most intense, doubly or triply charged, ions were sequentially isolated and fragmented in the linear trap by collision induced dissociation with an exclusion time of six seconds for the same ion species.

**Database analysis**

MS/MS data was analyzed using Thermo Proteome Discoverer (Thermo Scientific) with the Mascot search engine (Matrix Science, London, UK) and the UniProtKB/Swiss-Prot 55.3 database (selected for Homo sapiens, 19372 entries and trypsin as enzyme) allowing a fragment ion mass tolerance of 0.50 Da and a parent ion tolerance of 5.0 PPM. Iodoacetamide derivative of cysteine was specified as a fixed modification while oxidation of methionine was selected as a variable modification.

Protein identifications were accepted if they had greater than 95.0% probability and contained at least two identified unique peptides, which resulted in a maximal false discovery rate for protein identifications of 0.64%. Proteins that contained similar peptides and could not be differentiated based on MS/MS analysis alone were grouped.
